# Lab-on-a-Chip and Microfluidics Technologies for Nano Drug Delivery

**DOI:** 10.3390/bioengineering13030363

**Published:** 2026-03-20

**Authors:** Bochun Guo, Yuchao Zhao, Xunli Zhang

**Affiliations:** 1 School of Engineering, University of Southampton, Southampton SO17 1BJ, UK; 2 School of Biomedical Engineering, Faculty of Engineering, University of New South Wales, Sydney, NSW 2052, Australia; 3 School of Chemistry & Chemical Engineering, Yantai University, Yantai 264005, China

**Keywords:** lab-on-a-chip, microfluidics, nanomedicine, drug delivery systems, nanoparticle synthesis

## Abstract

Lab-on-a-Chip (LoC) and microfluidic technologies are rapidly reshaping the development pipeline for nano drug delivery systems (DDSs) by enabling precise control of physicochemical properties, high-throughput screening, and integrated biological evaluation within miniaturized platforms. This review synthesizes recent advances in microfluidic principles, fabrication strategies, and sensing modalities that facilitate continuous flow synthesis, real-time characterization, and adaptive formulation of nanoparticles. We highlight how LoC-enabled systems improve monodispersity, reproducibility, and tunability of liposomes, polymeric nanoparticles, and metallic nanocarriers, while providing powerful tools for assessing pharmacokinetics, drug release, and systemic responses using organ-on-chip (OoC) models. Emerging trends, including AI-driven autonomous optimization, stimuli-responsive materials, 3D-printed hybrid architectures, and self-powered portable devices, are discussed in the context of future integrated nano-pharmaceutics platforms. Despite existing challenges related to biocompatibility, standardization, data integration, and translation to industrial and clinical applications, the synergistic evolution of LoC engineering and nanomedicine holds transformative potential for personalized and next-generation therapeutic strategies.

## 1. Introduction

### 1.1. Background on Nano Drug Delivery Systems (DDSs)

**Figure 1:**
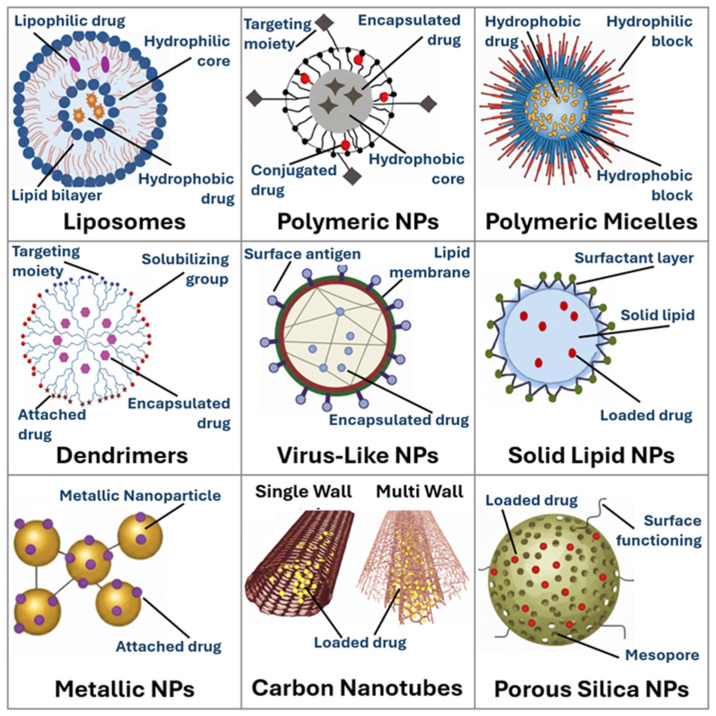
Schematic illustration of different types of nanoparticle (NP)-based drug delivery systems (DDSs).

Nano drug delivery systems (DDSs) are nanostructures, generally with at least one dimension in the 1–100 nm range, that serve as carriers to encapsulate or attach therapeutic agents for delivery to specific tissues or organs. They operate through mechanisms, including passive and active targeting, and enable controlled release, which minimizes systemic toxicity and maximizes therapeutic efficacy [1,2,3]. These nanostructures can take the form of polymeric nanoparticles, liposomes, dendrimers, micelles, solid-lipid nanoparticles, inorganic nanoparticles, and hybrid constructs [4,5,6]. Each of these has distinct advantages. For example, liposomes are biocompatible and versatile, whilst polymeric nanoparticles can be engineered for controlled degradation, and dendrimers offer precise, multivalent surface chemistry. Collectively, these systems enhance drug solubility, improve bioavailability, prolong circulation times, and reduce off-target toxicity through passive targeting with, e.g., enhanced permeability and retention (EPR) effect, or active targeting via ligand modification. Consequently, nano DDSs have, over the past decades, rapidly become a cornerstone of modern therapeutics, leveraging their unique physical and chemical properties to improve targeting, stability, and efficacy [7,8,9]. Figure 1 illustrates a range of drug delivery systems, including solid-lipid nanoparticles; liposomes; dendrimers; polymeric nanoparticles; polymeric micelles; virus-like nanoparticles; and inorganic nanoparticles such as metallic nanoparticles, carbon nanotubes and mesoporous silica nanoparticles.

Recent advances in nano DDSs increasingly focus on “smart” or stimuli-responsive nanoparticles, which release therapeutic cargo in response to internal triggers such as pH changes, redox gradients, or enzymatic activity, as well as external stimuli, including temperature, magnetic fields, or light [10,11,12,13,14]. Such stimulus-responsiveness enables site-specific drug release, thereby reducing systemic exposure and minimizing adverse effects. In particular, nanocarriers engineered for cancer immunotherapy employ immune-modulatory strategies to reprogram the tumor microenvironment (TME), thereby enhancing both the efficacy and selectivity of immunotherapeutic agents. By delivering immune stimulants, checkpoint inhibitors, or cytokines directly to the TME, these nanosystems can overcome immunosuppressive barriers, activate cytotoxic T-cell responses, and improve therapeutic outcomes [15,16,17,18,19].

Moreover, recent reviews have highlighted substantial progress in targeting cancer cell surfaces using ligand-functionalized nanocarriers, where biorecognition moieties such as folic acid, transferrin, and hyaluronic acid are employed to improve the specificity of nanoparticle binding to tumor-associated receptors [20,21,22]. This targeted approach enhances cellular uptake and tumor selectivity while minimizing off-target accumulation and systemic toxicity [23,24]. Another area of strong development lies in domain-specific applications. For instance, researchers have advanced nanomaterial-based ocular drug delivery systems to overcome physiological barriers in the eye, such as rapid drainage and membrane permeability, thereby enabling sustained and more efficient therapeutic delivery [25,26,27].

However, despite these advances, important challenges remain. For large-scale use, scaling up the production of nanoparticle-based DDSs while maintaining batch-to-batch reproducibility is difficult. Concerns also persist regarding long-term safety, immunogenicity, and regulatory pathways. In addition, nano-bio interactions (e.g., protein corona formation and immune recognition) can unpredictably influence pharmacokinetics and biodistribution [2,28,29]. To translate nanomedicine more broadly into clinical practice, these issues must be addressed through improved design, rigorous in vitro and in vivo testing, and strategic manufacturing processes.

### 1.2. Emergence and Evolution of Lab-on-a-Chip (LoC) Technology

**Figure 2:**
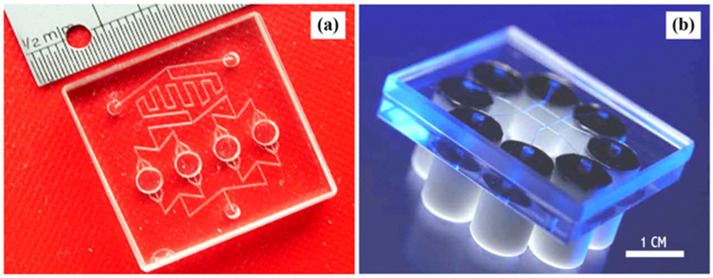
Lab-on-a-chip devices showing microfluidic channels, micromixers, and detection components. (**a**) Glass chip. (**b**) Glass chip with ceramic reservoirs and electrodes.

Lab-on-a-Chip (LoC) is a technology enabling the performance of macroscale laboratory operations on miniaturized devices [30]. The key feature of the LoC system is the microscale flow channel (with diameters between a few microns and hundreds of microns), where microfluidics is restricted to laminar flow, enabling the control and manipulation of fluid and reagent interaction [31,32]. Consequently, this miniaturization of chemical processes offers many fundamental and practical advantages in the realization of controllable, information-rich, high-throughput, and environmentally friendly methods for bio/chemical analysis and production [33]. Figure 2 shows two examples of LoC devices designed for conducting microscale reactions and on-chip analysis.

Rooted in microfluidics, LoC technology has evolved dramatically since its early days [34]. Initially derived from microelectromechanical systems (MEMS) engineering, early LoC platforms used rigid materials like silicon or glass to create channels and micro-chambers [35,36,37]. Over time, advances in soft lithography (e.g., using PDMS) [38,39]; polymer and paper microfabrication [40,41]; and, more recently, 3D printing have been applied to microfluidic device construction, lowering costs and increasing biocompatibility [42,43].

One of the most transformative advances in LoC technology has been the integration of droplet microfluidics, which enables the formation of highly monodisperse microscale droplets acting as individual microreactors [44,45,46]. This innovation has profound implications for nanoparticle synthesis, offering precise control over reaction environments, mixing dynamics, and mass transfer that are difficult to replicate in bulk systems. By tuning flow rates, droplet sizes, and mixing regimes, microfluidic systems can reproducibly produce nanoparticles with narrow size distributions, controlled loading efficiency, and tailorable surface properties [47]. Recent work demonstrates that droplet-based reactors allow for on-demand mixing control and rapid reaction kinetics, leading to enhanced particle uniformity and functionalization versatility for materials such as magnetic nanoparticles [48], silica nanoparticles [49], and lipid-based nanostructures [50]. Such precision and reproducibility remain largely unattainable in traditional batch synthesis, where mixing inefficiencies and variable kinetics often yield broad polydispersity and inconsistent product quality.

Parallel to synthesis applications, LoC platforms have progressed significantly in mimicking physiological environments, namely, organ-on-chip systems (e.g., tumor-on-chip, blood–brain barrier (BBB)-on-chip, and liver-on-chip) now recapitulate key aspects of organ microarchitecture, fluid flow, and cell–cell interactions [51,52,53]. These platforms offer powerful tools for preclinical testing of nanomedicines. For example, they enable real-time studies of nanoparticle transport, cellular uptake, toxicity, and pharmacodynamics in a controlled microenvironment, reducing reliance on animal models [54,55,56,57,58].

In the last couple of years, there has also been a surge in AI-enhanced microfluidic systems. Artificial intelligence has been demonstrated to automate and accelerate the design, operation, and high-throughput screening of microfluidic platforms [59,60,61]. Furthermore, micro- and nanoscale robotics (micro/nanorobots) have been applied in microfluidic environments as AI-enhanced biomedical micro/nanorobots for tasks such as targeted delivery, sensing, and actuation within microfluidic networks [61,62,63].

Overall, LoC has transitioned from a simple tool for miniaturizing laboratory processes to a sophisticated, multifunctional platform capable of not only synthesizing nanomaterials but also recapitulating biological systems, integrating real-time analytics, and harnessing machine intelligence for scalable, automated workflows. Table 1 compares the performance of LoC/microfluidic platforms with conventional batch systems for the synthesis of nano DDSs.

### 1.3. Scope and Organization of the Review

This review aims to synthesize the developments and emerging trends in how LoC technologies have advanced nano drug delivery research over the past decade. The Web of Science and PubMed databases were used as the primary bibliographic sources for identifying and retrieving relevant articles. The literature search was conducted using the following keywords: lab-on-a-Chip, microfluidics, nanomedicine, drug delivery systems, and nanoparticle synthesis. Articles identified through this search strategy were screened and selected for inclusion in this narrative review. Fundamentals of LoC and microfluidic technologies are first discussed, including principles; materials and fabrication; fluidic handling and control; and integration of analytical techniques. The current state-of-the-art is then articulated for the synthesis and characterization of nanoparticle-based DDSs using LoC and microfluidic technologies, highlighting on-chip characterization tools for monitoring nanoparticle size, stability and drug loading, together with progress in scaling up nano DDSs production.

Building on this foundation, the review next explores the synergistic interfaces between LoC systems and nano DDSs, including on-chip screening platforms, real-time monitoring of pharmacokinetics, and advanced organ-on-a-chip models that recapitulate physiological responses to nanocarriers. These sections underscore how microengineered environments enable predictive in vitro and in vivo simulations, thereby accelerating preclinical assessment.

The subsequent section critically examines current challenges and limitations, focusing on biocompatibility constraints; scaling and reproducibility issues; data integration hurdles; cost barriers; and the regulatory landscape. The aim is to contextualize existing gaps that must be addressed to achieve broader translational impact.

Finally, the review surveys emerging trends and future perspectives shaping the next decade of LoC-enabled nanomedicine, including AI-driven optimization, smart responsive nanomaterials, 3D-printed microfluidics, portable self-powered systems, and integrated nano-pharmaceutics platforms poised for clinical and pharmaceutical adoption. Together, these sections provide a structured roadmap designed to guide researchers, engineers, and clinicians toward the strategic development of next-generation LoC–nanomedicine technologies.

## 2. Fundamentals of Lab-on-a-Chip (LoC) and Microfluidic Technologies

### 2.1. Principles of LoC and Microfluidics

Lab-on-a-chip (LoC) systems rely on the physics of fluids at the microscale, where fluid dynamics differ markedly from that observed in conventional macroscopic systems due to the reduced dimensions and characteristic flow conditions [30]. Microfluidics typically involves the manipulation of very small fluid volumes (from microliters to picoliters) within microchannels whose characteristic dimensions range from tens to hundreds of micrometers. Under these conditions, the governing transport phenomena are strongly influenced by the relative balance between inertial and viscous forces, which is commonly described by the dimensionless Reynolds number (*Re*), which is defined as

(1) *Re* = *ρUL*/*μ*

where *ρ* is the fluid density, *U* is the characteristic velocity, *L* is the characteristic length scale, and *μ* is the dynamic viscosity.

*Re* provides a measure of the flow regime within the system. In microfluidic channels, the small length scales and moderate flow velocities typically result in very low Reynolds numbers (*Re* < 1), indicating that viscous forces dominate over inertial forces [30,72]. Consequently, fluid flow in these systems occurs in a laminar regime, where parallel fluid streams move along the channel with minimal transverse mixing. Unlike macroscopic systems, where turbulence can promote rapid homogenization of fluids, turbulence is essentially absent under microfluidic conditions. Instead, mixing between adjacent fluid streams occurs primarily through molecular diffusion across concentration gradients. While diffusion is effective over very short distances, it can be relatively slow when larger channel widths or higher flow rates are involved, which can limit mixing efficiency within microfluidic devices.

This diffusion-dominated transport behavior presents both opportunities and challenges in LoC system design. On one hand, the predictable and highly controlled laminar flow environment allows for precise manipulation of fluids, enabling reproducible chemical reactions, gradient formation, and controlled nanoparticle synthesis. On the other hand, the absence of turbulence necessitates deliberate design strategies to enhance mixing efficiency. Consequently, numerous microfluidic architectures have been developed to overcome diffusion limitations, including passive micromixers that introduce geometric features to promote chaotic advection, as well as active micromixers that employ external energy sources, such as acoustic, electrokinetic, or magnetic fields, to improve mixing under laminar flow conditions [73].

Another characteristic feature of microfluidic systems is their high surface-to-volume ratio, which significantly enhances surface interactions, improves mass and heat transfer, and enables rapid equilibration. These properties lend LoC devices their sensitivity and precision: the short diffusion path lengths enable rapid mixing, reaction kinetics can be tightly controlled, and gradients (chemical, thermal, and shear) can be precisely established [74,75]. The combination of efficient transport phenomena and precise microscale control allows LoC platforms to outperform traditional macroscale systems in terms of speed, reproducibility, and energy efficiency [76].

Beyond simple pressure-driven flow, many LoC platforms exploit alternative transport modes. For example, electrokinetic phenomena such as electroosmotic flow (EOF) can be used to drive fluid motion by applying an electric field across microchannels [77,78]. EOF arises from the electrical double layer at solid–liquid interfaces and permits flow without mechanical pumps, which is highly valuable for miniaturization [79,80]. Additionally, droplet microfluidics is another paradigm as discussed above. By generating discrete droplets (microreactors) in a carrier fluid, one can compartmentalize reactions, avoid cross-contamination, and achieve extremely high throughput. These droplets can be manipulated (merged, split, and mixed) to program complex reaction sequences [45,49].

Recent perspectives suggest that next-generation microfluidics will go even further, integrating more sophisticated transport phenomena and harnessing the unique scaling laws of microscale flows to better mimic physiological conditions [81].

### 2.2. Materials and Fabrication Techniques

#### 2.2.1. Materials

The choice of material and fabrication method is central to the performance, biocompatibility, and scalability of LoC devices [37]. Commonly used materials include silicon, glass, metals, and polymers. Traditionally, polydimethylsiloxane (PDMS) has been the gold-standard material due to its optical transparency, flexibility, gas permeability, and ease of fabrication via soft lithography. However, PDMS has limitations; it can absorb hydrophobic molecules, swell in organic solvents, and age over time, which can affect long-term device performance [82].

In addition, several intrinsic properties of PDMS raise biocompatibility challenges. Notably, the hydrophobic nature of PDMS promotes non-specific adsorption and absorption of proteins, small molecules, and hydrophobic drugs, potentially altering analyte concentrations and affecting experimental outcomes [83,84]. This absorption can be particularly problematic in pharmacological studies or organ-on-chip systems, where accurate dosing and molecular transport are essential. Additionally, PDMS surfaces often require surface modification or plasma treatment to improve hydrophilicity and facilitate cell adhesion, yet these treatments can suffer from hydrophobic recovery, leading to unstable surface properties over time [85]. Furthermore, unreacted oligomers or leachables from incompletely cured PDMS may influence cellular responses, highlighting the need for careful material preparation and validation.

#### 2.2.2. Fabrication Techniques

Photolithography. Traditionally, LoC devices have been fabricated using photolithography, a method that originates from the MEMS manufacturing process. The process starts with coating a silicon or glass substrate with a photoresist layer, which is then selectively exposed to UV light through a patterned photomask. The exposed regions undergo chemical changes (etching) and are subsequently developed to create the desired microscale channel patterns on the substrate [30].

Soft lithography. This is a widely used fabrication technique for LoC devices that relies on elastomeric materials, most commonly PDMS [39]. The process typically involves casting liquid PDMS onto a microstructured master mold, curing it to form an elastomeric replica, and then bonding the patterned PDMS layer to a glass or polymer substrate to create enclosed microfluidic channels.

Injection molding. This is a high-throughput fabrication technique widely used for the mass production of polymer-based LoC devices. In this process, molten thermoplastic material is injected into a microstructured mold cavity, where it cools and solidifies to replicate the desired microfluidic features before being ejected as a finished component [37].

3D printing. It should be noted that, recently, there has been rapid growth in additive manufacturing (3D printing) for microfluidic device fabrication. In particular, stereolithography (SLA), digital light processing (DLP), two-photon polymerization (2PP), and extrusion-based approaches are now commonly used to build microfluidic architectures that are difficult or impossible with traditional soft lithography [86,87].

A critical advance is the development of tenable resins for SLA that mimic the elasticity of PDMS. For instance, newly formulated resins based on PEGDA-co-PEGMEMA can produce printed elastomeric components (e.g., valves, pumps) with moduli close to PDMS, while retaining printability and biocompatibility. Moreover, 3D printing enables multimaterial fabrication, hierarchical structures, and freeform, non-planar geometries. A recent critical review highlights how 3D-printed microfluidics now integrate microstructures, valve networks, and sensing arrays in fully 3D architectures [88]. Light-based 3D printing of molds for PDMS soft lithography is also evolving, where new approaches employ vat photopolymerization to generate master molds at higher resolution and with more complex features [89].

**Figure 3:**
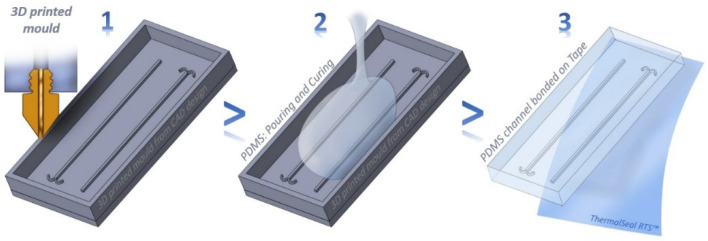
Schematic of the workflow for the fabrication of a microreactor. (1) Low-cost 3D printing of the mold. (2) PDMS pouring and curing. (3) Sealing on the adhesive tape. [42] (Adapted with permission from Elsevier.)

The combination of the above techniques has also proved to be advantageous. For example, Cristaldi et al. [42] developed a cost-effective and easy-to-perform fabrication method for the generation of optically transparent, continuous-flow reactors. The method combines 3D printing of master molds with the sealing of the PDMS channels’ replica using a pressure-sensitive adhesive tape. Figure 3 schematically shows the workflow of the fabrication process.

### 2.3. Fluid Handling and Flow Control Mechanisms

#### 2.3.1. Pressure-Driven Flow (PDF)

The most straightforward and widely used approach for driving flow in microfluidic systems involves the use of external pumps, such as syringe, peristaltic, or pneumatic pumps, to drive fluids through microchannels. The resulting pressure differential across the channel generates a predictable laminar flow regime. In such systems, the velocity profile of the fluid is parabolic across the channel cross-section, a hallmark of pressure-driven flow at low Reynolds numbers [90]. However, this non-uniform flow distribution can lead to shear-dependent transport phenomena, posing challenges for the precise manipulation and control of reagents or microparticles along the microchannel [91]. Further innovations, such as feedback-controlled syringe pumps and hybrid electroosmotic-pressure systems, have been developed to mitigate flow instability and improve accuracy in particle handling [92].

#### 2.3.2. Electroosmotic Flow (EOF)

Electroosmotic flow (EOF) is particularly advantageous in microfluidic systems, where minimizing mechanical components and eliminating pulsatile motion are desirable. In EOF, an applied electric field across the microchannel induces motion of the mobile ion layer (electric double layer) near the channel walls, which in turn drags the bulk fluid through viscous coupling [77,93]. This method enables highly precise and steady flow control without the need for moving parts, providing vibration-free operation and exceptional reproducibility [78,94].

Compared to the more common pressure-driven microflows, EOF produces an almost plug-like velocity profile across the channel cross-section, minimizing shear stress and ensuring uniform residence times for suspended analytes [94]. This uniformity makes EOF particularly effective in analytical applications such as electrophoresis, sample injection, and on-chip separations. However, EOF can also introduce limitations such as Joule heating, which may distort flow uniformity, and electrophoretic demixing of charged analytes under high electric fields [95].

#### 2.3.3. Passive Flow Control Methods

Capillary-driven flow is one of the most important passive mechanisms in microfluidic systems, especially in paper-based microfluidics, where liquid motion is governed by surface tension and capillary forces rather than external pumps [96,97]. The inherent porosity and hydrophilicity of paper substrates enable spontaneous imbibition of liquids through interconnected pores, allowing precise and power-free fluid handling [98]. By tuning material properties, such as pore size, contact angle, and surface energy, flow velocity and transport length can be modulated, making these systems ideal for low-cost diagnostics and point-of-care assays [98].

Beyond porous substrates, microstructural design plays a critical role in regulating passive flow. Fluidic resistors, channel constrictions, and hydrophobic/hydrophilic patterning can introduce spatial control over pressure and velocity distributions, effectively functioning as microvalves or mixers without any active components. Such passive elements enable sophisticated flow manipulation. For instance, staggered hydrophobic barriers can delay or synchronize multiple streams, while gradient surface modifications can guide fluid directionality and partition analytes for multiplexed detection. Recent modeling work has also demonstrated that careful design of geometric and chemical gradients allows predictable capillary flow rates and stable front propagation even in closed microchannels [99].

#### 2.3.4. Droplet Microfluidics

Droplet-based microfluidic systems exploit discrete droplets as isolated reaction compartments, enabling precise control of microscale chemical and biological processes. Droplet generation typically occurs at geometric junctions such as T-junctions or flow-focusing configurations, where immiscible fluids meet under controlled flow conditions, causing periodic pinch-off of droplets [45,49,50]. In these systems, droplet size and uniformity are primarily governed by flow-rate ratios, interfacial tension, and channel geometry. More advanced strategies introduce valve-assisted or pressure-based control to enhance monodispersity and droplet generation stability, as demonstrated by recent work employing negative pressure or pneumatic actuation [47,100].

Once formed, droplets can be manipulated dynamically within microchannels through passive or active methods. Passive manipulation relies on microchannel geometries or interfacial interactions to induce controlled splitting, merging, or coalescence. For example, carefully designed bifurcations or constrictions can split droplets evenly, while hydrophilic patches promote coalescence at designated points. Active manipulation, in contrast, leverages external stimuli such as electric fields, pneumatic valves, or acoustic waves to achieve programmable droplet behavior [101]. Techniques like electrowetting-on-dielectric (EWOD) and dielectrophoresis (DEP) allow for droplet transport and merging with submillisecond precision, while surface acoustic wave (SAW)-based systems enable non-contact, high-frequency droplet actuation [102].

### 2.4. Integration of Detection and Sensing Modules

A defining strength of LoC systems lies in their capacity to integrate in situ detection and sensing modules, enabling real-time quantitative analysis of fluids, cells, and molecular species within the microscale environment. The detection methods include optical, electrochemical, and mass spectrometric techniques, each offering unique advantages depending on the analyte and application, as discussed below.

#### 2.4.1. Optical Sensors

Optical detection methods, such as fluorescence, absorbance, and surface plasmon resonance (SPR), allow highly sensitive, label-based or label-free detection and are widely used for biomolecular assays and single-cell studies. This can be realized by aligning optical fibers or waveguides with microchannels [103] or coupling with microscopic optics [104]. For instance, a recent work reviews microfluidic platforms integrated with fiber-optic sensors for on-site detection of biomolecules, leveraging the small footprint and high sensitivity of fiber optics [105].

More advanced devices use two-photon polymerization or other high-resolution printing methods to co-fabricate microfluidic channels and optical waveguides or photonic structures, allowing for evanescent field sensing, high-sensitivity spectroscopic measurement, and even mid-infrared sensing [106].

#### 2.4.2. Electrochemical Sensors

Electrochemical sensors are widely integrated into LoC systems, where microfabricated electrodes are embedded within microchannels or patterned on-chip substrates to detect electroactive species such as ions, metabolites, and redox-active molecules. These electrodes, commonly composed of gold, platinum, carbon, or conductive polymers, can be fabricated through photolithography, screen-printing, or inkjet printing and seamlessly interfaced with microfluidic flow networks [107]. Integration of these sensing elements allows for in situ, label-free detection with high sensitivity and rapid response times, enabling real-time monitoring of biochemical reactions, microorganism activity, or environmental pollutants within microscale volumes [107,108].

The versatility of electrochemical transduction, encompassing amperometric, potentiometric, conductometric, and impedimetric modes, allows for customization for diverse analytical needs. For instance, amperometric sensors are commonly used to detect glucose or neurotransmitters via oxidation–reduction reactions, while impedance-based sensors can probe cell adhesion, membrane integrity, or biofilm formation on-chip [108,109]. Recent advances have incorporated nanostructured electrodes and graphene-based materials to enhance electron transfer and surface area, thereby improving detection limits to the nanomolar range [110,111].

#### 2.4.3. Spectrometric-Based LoC Sensing Platforms

Spectrometric detection methods, especially Raman spectroscopy and absorbance/optical spectrophotometry, have become increasingly integrated into modern LoC platforms, enabling highly sensitive, label-free, and chemically specific on-chip analysis. By combining microfluidics with surface-enhanced Raman scattering (SERS), it can greatly improve detection sensitivity relative to conventional fluorescence or UV/vis absorbance, overcoming limitations arising from low pathlength and small sample volumes in microscale devices [112,113]. For example, Li et al. [114] developed a microspectrometer-integrated microfluidic chip for real-time monitoring of protein concentration, achieving up to an 833-fold increase in local concentration (from ~10 nM initial) within ~20 min under flow, demonstrating that on-chip spectrometric detection can combine both enrichment and analysis in one device.

Beyond static detection, spectrometric-based microfluidic platforms are increasingly supporting dynamic, high-throughput, and high-sensitivity applications. Notably, platforms coupling microfluidics with mass spectrometry (MS) have opened new frontiers in microproteomics: integrated workflows now allow for sample preparation, digestion, labeling, and direct injection into LC-MS, enabling in-depth proteomic profiling of scarce samples or even single cells [115]. By coupling with a microspectrometer, Zmijan et al. demonstrated in situ microspectroscopic monitoring of the dynamic process of nanoparticle formation, with their rapid changes in size and shape along microfluidic channels through absorbance measurements [104]. Furthermore, a trend toward multimodal detection is evident for multiplexed, on-chip diagnostics that maximize both sensitivity and robustness [116].

## 3. LoC-Based Synthesis and Characterization of Nanoparticle DDSs

### 3.1. Continuous Flow Synthesis of Nano DDSs (Liposomes, Polymeric NPs, Metallic NPs)

**Figure 4:**
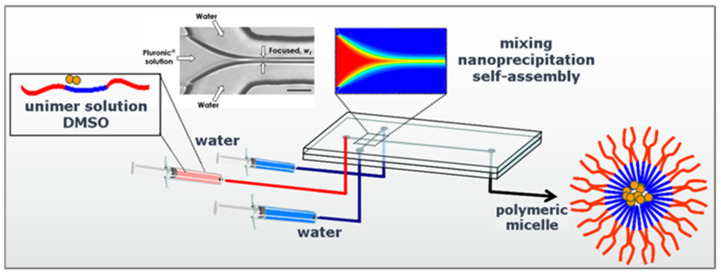
Continuous flow microfluidic reactor for synthesizing polymeric micelles. Inserts: Microscopic image (scalebar: 100 μm) and CFD simulation in the hydrodynamic flow focusing region. Arrows show the flow direction and the flow focusing region.

Continuous flow microfluidic platforms are increasingly used for the manufacture of nanoparticle (NP) drug delivery systems due to their superior reproducibility, tunable control over particle properties, and potential for scalable production. In contrast to conventional bulk synthesis methods, microfluidic systems enable rapid and highly controlled mixing of solvents and reagents within micrometer-scale channels, allowing for precise regulation of nanoparticle nucleation and growth. This level of control reduces batch-to-batch variability and improves the uniformity of nanocarrier formulations, which is particularly important for pharmaceutical translation and regulatory approval. As a result, microfluidic technologies have emerged as powerful tools for producing lipid-based nanocarriers, polymeric nanoparticles, and hybrid nanostructures with highly consistent physicochemical characteristics (Figure 4) [31,117,118].

For example, microfluidic synthesis of lipid-based nanoparticles, such as liposomes and lipid nanoparticles (LNPs), is commonly achieved using hydrodynamic flow focusing or staggered herringbone micromixers, which promote rapid solvent exchange and controlled self-assembly of lipid molecules. These methods enable tight control over critical formulation parameters, including particle size, polydispersity index (PDI), and drug encapsulation efficiency, while also allowing systematic adjustment of flow rates, solvent ratios, and lipid concentrations to fine-tune nanoparticle characteristics [31,117,118]. Such precise control has been instrumental in the development of modern lipid nanocarriers used in nucleic acid delivery and other therapeutic applications.

When biodegradable polymers such as PLGA (poly(lactic-co-glycolic acid)) are incorporated into microfluidic continuous flow systems, hybrid nanoparticle architectures can also be produced with high reproducibility. For instance, microfluidic synthesis has enabled the fabrication of hybrid “nanolipomer” structures consisting of a PLGA polymeric core surrounded by a PEGylated lipid shell. Using optimized high-flow microfluidic conditions, these nanostructures can be produced at flow rates of up to approximately 12 mL min^−1^, yielding particles with narrow size distributions around ~100 nm and high encapsulation efficiencies for hydrophobic drugs such as curcumin. Importantly, these studies demonstrate that microfluidic synthesis parameters optimized at a small scale could be translated to higher throughput production systems, highlighting the potential of continuous flow microfluidics for scalable nanomedicine manufacturing [119,120,121].

Metallic nanoparticles (such as gold, silver and iron oxide) have also been synthesized in microreactors where microfluidic mixers allow for rapid nucleation and growth under controlled mixing times, enabling precise size control. More mixing approaches, including passive mixing and active methods with electrical or acoustic actuation and integrated feedback control, have been investigated to enhance the process controllability [64].

Further to continuous flow synthesis, modern microfluidic platforms are increasingly being designed to integrate downstream processing modules such as post-synthetic functionalization, purification, buffer exchange, and product concentration, all within a unified continuous flow architecture [117,122]. Post-synthetic functionalization steps, including ligand conjugation, surface coating, and PEGylation, can be precisely controlled on-chip through segmented or droplet-based flow regimes that ensure homogeneous reaction environments and minimize reagent consumption [123]. For post-synthesis functionalization and ligand insertion after forming liposomes, a secondary microfluidic mixing module can be used to insert ligands (e.g., peptides and PEG-lipids) or drug–lipid conjugates under continuous flow, all while maintaining tight residence times and uniformity [117].

### 3.2. In Situ Characterization Tools (Spectroscopy, Imaging, Scattering)

Characterizing nanoparticles in situ (i.e., within or immediately after synthesis in the chip) is critical to maintain control over quality. Microfluidic devices can interface directly with analytical modalities for real-time monitoring and on-chip evaluation [124], including optical, electrochemical, and fluorescence-based detections. These detections can be carried out off-chip or on-chip or even be integrated into chips, as discussed above. For example, one powerful method examined is synchrotron X-ray scattering integrated into microfluidic chips. In a study by Martín-Asensio et al. [125], a 3D-printed microfluidic chip was filled with a Matrigel-like tumor matrix and combined with synchrotron X-ray scattering to map nanoparticle diffusion in a biomimetic microenvironment, without requiring fluorescence labels that can perturb NP behavior.

Optical methods have also been explored, e.g., using opto-fluidic force induction, where particles flowing through a laser beam in a microchannel experience size-dependent optical forces, enabling real-time size distribution measurement with single-particle sensitivity [126]. Another useful approach is diffusiophoresis-based manipulation. By imposing gradients of salt (or other solutes) in microchannels, charged colloids (e.g., liposomes and beads) can be driven and focused. This can also provide a measurement of surface charge or zeta potential under flow [127].

More generally, microfluidic platforms can integrate fluorescence, absorbance, or Raman spectroscopy detectors for online monitoring of drug loading, aggregation, or chemical changes, especially when combined with microfabricated waveguides or optical fibers. Reviews of microfluidic synthesis of nanoparticle DDSs emphasize these trends [64,128].

### 3.3. Controlled Drug Loading On-Chip

Controlled drug loading to nanoparticles on microfluidic chips offers many advantages. As discussed above, the mixing regime in microfluidic systems can be precisely tuned to influence nucleation, growth, and drug incorporation [73]. For liposomes, drug loading can be achieved during the formation step by co-fluxing the aqueous drug phase and the lipid phase or via post-synthesis drug insertion [31,118]. Furthermore, hybrid nanoparticles (e.g., polymer–lipid) synthesized in microfluidics can optimize drug loading by varying the ratio of polymer to lipid, flow rates, and solvent polarity and even by adding functional excipients, resulting in high encapsulation efficiency and stable formulation [119].

Recent advancements in inline purification technologies, including microfluidic dialysis, buffer exchange, and solvent removal systems, have significantly enhanced the efficiency and integration of drug manufacturing workflows [129,130]. These strategies enable the real-time removal of free drugs, unreacted precursors, solvents, and by-products immediately after synthesis, reducing downstream processing steps and improving overall product quality. By incorporating these purification modules into microfluidic or continuous flow systems, researchers can now directly couple synthesis and drug loading, achieving an integrated, continuous manufacturing pipeline that minimizes batch-to-batch variability and accelerates process scalability [117].

### 3.4. Scalability of Nano DDSs Production

Scalability remains a major barrier to translating microfluidic-based nanoparticle synthesis from laboratory prototypes to clinical or industrial manufacturing. This challenge arises primarily from the intrinsic microscale nature of microfluidic systems. While offering precise control and superior mixing compared to conventional macrofluidic or bulk synthesis methods, these systems inherently limit volumetric throughput and scale-up potential.

Recent advances, however, are making this more feasible. As noted, a universal microfluidic platform was demonstrated that can vary flow rates from 0.1 to 75 mL/min, enabling both small-scale research production and high-throughput industrial manufacturing without sacrificing mixing performance or nanoparticle quality [131]. This was realized using a coaxial flow design with a vortex-generating microstructure, allowing for both small exploratory formulation and industrial-level production of NPs (lipid, polymer, and polyplex) [131]. Another key strategy commonly employed is through the parallelization of microfluidic channels. It has been demonstrated that microfluidic architectures using multiple parallel mixers or channels can substantially increase throughput while maintaining tight control over particle size and encapsulation metrics [128].

Finally, microfluidics-based automated systems are being deployed for clinically relevant NP production. For instance, a multichannel donut-ring microfluidic design was used to produce mRNA-loaded lipid nanoparticles consistently and reproducibly, supporting gene delivery applications [132]. Furthermore, to fully industrialize microfluidic NP production, integration of synthesis, purification, monitoring and process control with machine learning/feedback systems (“intelligent microfluidics”) has been explored [59,60,64].

### 3.5. Clinically Relevant Nanomedicines Produced Using Microfluidics

The clinical success of nanomedicines has demonstrated the therapeutic potential of nano drug delivery systems in improving pharmacokinetics, reducing systemic toxicity, and enhancing targeted drug accumulation. One of the most widely cited examples is Doxil^®^, a PEGylated liposomal formulation of doxorubicin approved for the treatment of ovarian cancer, multiple myeloma, and Kaposi’s sarcoma [133]. Doxil^®^ significantly alters the pharmacokinetic profile of doxorubicin by prolonging circulation time and enabling enhanced tumor accumulation via the enhanced permeability and retention (EPR) effect. These clinically validated nanomedicines provide an important benchmark for the development of next-generation formulations fabricated using advanced technologies such as microfluidics.

Compared with conventional bulk preparation methods, microfluidic mixing allows for rapid nanoparticle self-assembly, leading to uniform particle sizes, narrow polydispersity, and improved encapsulation efficiency. For example, microfluidic rapid mixing has been successfully used to fabricate doxorubicin-loaded lipid nanocarriers with high drug-loading capacity and controlled release profiles, demonstrating comparable or improved therapeutic performance relative to conventional liposomal systems [134]. Similarly, microfluidic approaches have been used to produce temperature-sensitive liposomes capable of efficiently loading doxorubicin (>80% encapsulation efficiency) with controlled release triggered by mild hyperthermia [135].

Beyond laboratory-scale demonstrations, microfluidic manufacturing strategies are increasingly being explored for scalable production of clinically relevant liposomal formulations, including Doxil-like compositions consisting of hydrogenated phosphatidylcholine, cholesterol, and PEGylated lipids. Recent studies have shown that design-of-experiment optimization within microfluidic systems can enable robust, scalable production of monodisperse liposomes meeting pharmaceutical quality standards, highlighting the potential of microfluidics to bridge the gap between laboratory nanomedicine development and industrial manufacturing [136]. These advances demonstrate that microfluidic technologies can support the translation of nanomedicine formulations from research settings to clinically relevant production platforms.

## 4. Synergy Between LoC and Nano DDSs

### 4.1. On-Chip Screening and Optimization of Formulations

One of the most powerful synergies of LoC systems with nano DDSs lies in on-chip formulation screening and optimization. By integrating microfluidic synthesis and organ-on-chip (OoC) models, we can rapidly test how different NP parameters (such as size, size distribution, surface chemistry, and ligand density) affect biological interactions under physiologically relevant flow conditions. For example, tumor OoC devices have been used to evaluate the performance of polymeric nanocarriers synthesized via hydrodynamic focusing or micromixers, enabling detailed, dynamic assessment of NP uptake, penetration into tumor compartments, and cytotoxic effects [137].

Similarly, organ-on-chip platforms can facilitate the tuning of NP formulations in a more predictive environment than static cell culture. For instance, simulating the perfusion, shear stress and gradients of nutrients or oxygen can provide a more realistic assessment of how candidate nanomedicines behave in vivo. In addition, OoC systems have been used to assess NP toxicity, targeting, and accumulation, demonstrating a more accurate approach than traditional models [52,124,138].

Overall, the application of on-chip screening and optimization of formulations can assist in the acceleration of design cycles. Instead of synthesizing batches, testing in standard in vitro assays, and then iterating, the microfluidic integration approach enables real-time feedback on biological efficacy, which can feed directly back into microfluidic synthesis parameters to refine NP design.

### 4.2. Real-Time Monitoring and Control of Pharmacokinetics and Drug Release

By integrating real-time monitoring capabilities, LoC and OoC systems can dynamically characterize pharmacokinetics (PK) and nanoparticle (NP) drug release within microphysiological environments. With embedded biosensors, such as transepithelial electrical resistance (TEER) electrodes, fluorescence-based probes, and on-chip mass spectrometry modules, these systems enable continuous measurement of NP transport, accumulation, and drug-release kinetics over time [139,140]. Such integrated monitoring provides a mechanistic understanding of NP behavior under physiological flow, including rates of tissue penetration, cellular uptake, and spatial–temporal drug payload release [141,142].

Advanced OoCs platforms have also been used to observe NP-mediated responses under flow conditions that mimic physiological circulation. This enables more accurate extrapolation of in vitro PK to in vivo behavior. The development of OoC technology not only refines predictions of drug distribution but also provides control over release, e.g., by triggering release in specific compartments [143,144].

Moreover, miniaturized devices for on-demand drug release are being developed. One recent study by Zhou et al. [145] used a microfluidic chip coupled with a GHz-frequency ultrasonic resonator to actuate an elastic valve, enabling rapid, controllable (millisecond-scale) drug release under electronic or magnetic control. Such systems could be incorporated into LoC-nano DDS platforms, offering precise spatiotemporal dosing in micro-tissues.

### 4.3. Organ-on-a-Chip (OoC) Models for Systemic Response to Nano DDSs

**Figure 5:**
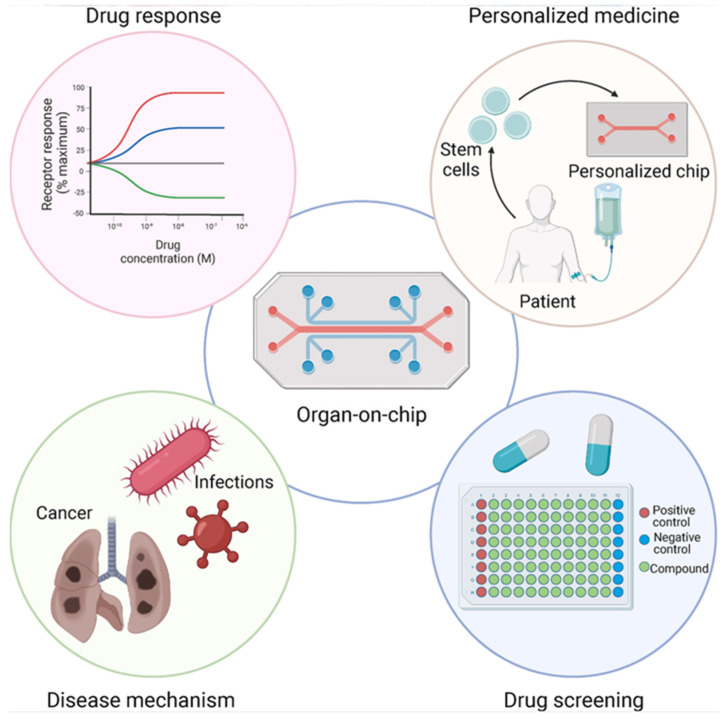
Schematic diagram depicting the multifaceted opportunities offered by OoC technology for drug response, personalized medicine, elucidation of disease mechanisms, and drug screening [146].

Beyond characterizing the physicochemical properties and pharmacokinetics of nano DDSs, OoC models provide a microengineered means to recapitulate organ-level architecture, function, and physiological barriers, thereby serving as robust platforms for investigating systemic responses to nano DDSs. These platforms have been developed to emulate a wide range of human organs, including the lung, liver, kidney, gut, heart, brain, and tumor microenvironment. In the context of nanomedicine, OoC devices facilitate the study of NP interactions with barrier tissues (e.g., endothelium and epithelium), immune cells, and dynamic flow conditions in multi-organ configurations, as illustrated in Figure 5 [146].

For instance, Stavrou et al. [52] examined how OoC systems are employed to model NP–tissue interactions, toxicity, and accumulation, capturing complex phenomena such as endothelial adhesion, translocation across biological barriers, and shear-stress-induced behaviors. Furthermore, OoC platforms have been increasingly utilized to evaluate the toxicity and therapeutic efficacy of nanomedicines, demonstrating that these microphysiological models replicate mechanical forces, fluid dynamics, and intercellular communication far more faithfully than conventional two-dimensional cultures or animal models [147]. For example, liver-on-chip and lung-on-chip devices can be used to investigate nanocarrier uptake, inflammatory responses, and cytotoxic effects under dynamic flow conditions that better mimic in vivo environments. A liver-on-chip model developed by Jang et al. [148], consisting of rat, dog, or human hepatocytes; endothelial cells; Kupffer cells; and stellate cells, confirmed the mechanism of action of several known hepatotoxic drugs and experimental compounds. The multispecies chip also enabled the identification of species-specific differences in drug metabolism and toxicity.

Moreover, the emergence of multi-organ or “body-on-a-chip” systems has enabled the investigation of multi-tissue pharmacodynamics, including NP or drug transit across organ barriers, metabolite formation, and downstream tissue responses. For example, a recent three-organ microfluidic model integrating gut, vascular, and neural compartments successfully demonstrated real-time monitoring of toxicity and metabolite transport, highlighting the capacity of interconnected OoC platforms to recapitulate organ interplay and systemic nanomedicine responses in vitro [149,150].

### 4.4. In Vitro and In Vivo Simulation On-Chip

In vitro simulation alone may not fully recapitulate in vivo complexity; however, LoC platforms combined with nano DDSs can bridge the gap by providing physiologically relevant simulation of in vivo behavior. By combining microfluidics, 3D tissue architectures, and controlled microenvironments, OoC devices can mimic absorption, distribution, metabolism, excretion (ADME), and toxicity processes in a highly controlled setting. For example, microfluidic OoC systems have been used to simulate barrier functions (like the blood–brain barrier and gut epithelium), helping to predict nanoparticle permeability, retention, and clearance [138].

**Figure 6:**
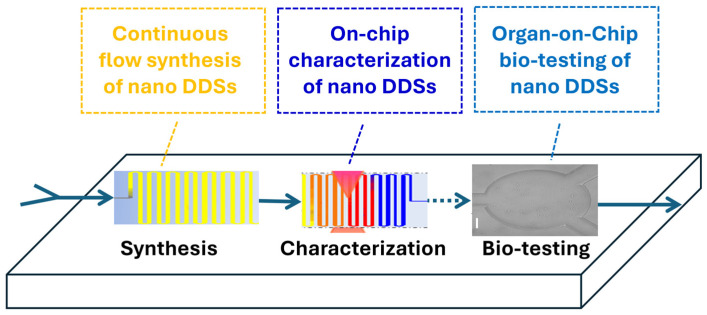
Microfluidics-based nanoparticle synthesis workflows and integrated LoC–OoC platforms.

As discussed above, microphysiological systems are increasingly being designed with multi-organ connectivity to simulate systemic transport of NPs, e.g., by linking a liver organ chip (for metabolic processing) to a downstream target organ chip to study both drug activation and toxicity. These multi-organ OoC models have been demonstrated to be crucial for translating nano DDS findings to the whole-body context, reducing reliance on animal models and improving predictive accuracy [144,149]. Coupling these systems with real-time analytical readouts (e.g., mass spectrometry, optical sensors, and electrical readouts) further strengthens their predictive power, offering a way to simulate not just steady-state exposure but dynamic, dose–response, and pharmacokinetic profiles that approximate in vivo conditions [143]. Figure 6 depicts schematic illustrations showing microfluidics-based nanoparticle synthesis workflows and integrated LoC–OoC platforms.

## 5. Current Challenges and Limitations

### 5.1. Biocompatibility and Scaling Issues

Despite these advancements, combining LoC microfluidics with nano DDSs still faces notable biocompatibility and scale-up challenges. One key issue is associated with the materials commonly used in microfluidics (e.g., PDMS) that can leach uncured oligomers or absorb hydrophobic drugs or proteins, potentially affecting biological compatibility and reproducibility. Additionally, when scaling up microfluidic synthesis for clinical or commercial production, the throughput required for therapeutic quantities of NPs often exceeds the capabilities of conventional microfluidic systems, as their small channel dimensions typically limit production volume. As pointed out by Shen et al. [151], although microfluidic systems offer excellent control over NP properties, producing the hundreds of grams needed for preclinical or clinical studies remains challenging.

To address these limitations for scale-up, researchers have begun exploring strategies such as parallelization of microfluidic channels, scaling-out approaches, and continuous-flow manufacturing systems, which allow multiple microreactors to operate simultaneously and increase overall productivity [34,119,131]. However, maintaining uniform mixing conditions and consistent NP characteristics across large-scale systems can still be technically demanding. Consequently, bridging the gap between the high precision of laboratory-scale microfluidic synthesis and the high throughput required for industrial production remains a key challenge for translating microfluidic nanoparticle fabrication into clinically and commercially viable drug delivery platforms.

Furthermore, the purification method used after microfluidic synthesis, such as centrifugation versus tangential flow filtration (TFF), can influence the biocompatibility of the resulting NPs. For example, Shaw et al. [152] found that PLA NPs made by microfluidics and purified by TFF exhibited different macrophage uptake, protein corona profiles, and biodistribution compared to particles purified by centrifugation.

In addition, the long-term safety of nano DDSs is another dimension to consider. Even if the initial formulation is biocompatible, nano–bio interactions (e.g., protein adsorption and immune recognition) in vivo can still be difficult to predict and replicate in microfluidic or small-scale systems. Microfluidic-produced NPs must be rigorously tested in biological systems to ensure they do not provoke unexpected toxicity or immunogenicity. As noted by Huang et al. [153], while microfluidic synthesis provides precision, there are still major challenges and future directions in ensuring nanomaterials remain safe and robust in biological environments.

### 5.2. Limited Physiological Relevance of Chip-Based Models

Microfluidic and lab-on-a-chip technologies have demonstrated precise control over the nutrient gradients, hydrodynamic shear forces, and spatial distribution of cells on-chip. However, significant limitations still remain, especially in modeling the real physiological environment of the human body. Physiological processes are highly dynamic and multi-level, involving blood flow, immune responses, neural regulation, and the spatiotemporal coordination of diverse biochemical signals. However, many contemporary chip-based models can reproduce only isolated local characteristics. In particular, these models struggle to simultaneously integrate inter-organ interactions and systemic feedback, and, therefore, cannot fully recapitulate physiological homeostasis or stress responses in living organisms [154].

In addition, the complex three-dimensional tissue architectures, extracellular matrix heterogeneity, and dynamic mechanical stimuli present in the in vivo microenvironment are often oversimplified at the chip level, which may alter cellular behavior and responses to nanopreparations [155]. In cancer- and inflammation-related studies, microchip models can simulate local microenvironmental conditions such as hypoxia and altered vascular permeability; however, accurately reproducing immune cell recruitment, systemic inflammatory signaling, and long-term pathological progression remains challenging [156]. Consequently, from the perspective of whole-body physiology, chip-based models generally represent a “simplified reconstruction” of selected physiological elements and cannot fully capture the complexity of living systems.

### 5.3. Standardization and Reproducibility

One of the most persistent obstacles to LoC and nano DDS integration is the lack of standardization, as current developments are largely based on individually customized designs and fabrication processes for research and development purposes [157]. Consequently, these microfluidic devices vary widely in materials (e.g., PDMS and thermoplastics), fabrication techniques, and connector configurations, complicating reproducibility across laboratories and hindering scale-up for manufacturing. For example, the diversity in material choices (e.g., PDMS, PMMA, and polycarbonate) presents significant challenges in ensuring consistent performance when transitioning from prototyping to mass production [158]. Furthermore, as noted by Gurkan et al. [81], inconsistent design choices and non-standard fabrication protocols remain major barriers to the clinical and industrial translation of microfluidic technologies.

Operational robustness is also a concern for microfluidic systems as they are sensitive to small variations in channel dimensions, surface chemistry, and fluidic connections. In a systematic study by Ducree et al. [159], failure modes such as bubble formation, misalignment, and variation in tolerances were examined during manufacturing and operation, directly impacting reproducibility. Moreover, toward clinical and/or industrial applications, the lack of standards for performance metrics (e.g., acceptable tolerances for flow rates, droplet sizes, and device-to-device variation) can significantly slow down processes for validation and regulatory approval.

### 5.4. Data Integration and Analytical Complexity

Integrating real-time data from microfluidic systems (e.g., flow rates, sensor outputs, and particle characteristics) into a coherent analytical framework remains a non-trivial challenge. As microfluidic NP synthesis grows increasingly complex, particularly within continuous manufacturing contexts, there is a rising demand for “intelligent microfluidics” that incorporates feedback control, machine learning, and automated analytics. Implementing these advanced approaches adds new layers of capability, enabling robust real-time measurement, stable sensing performance, and computational models that can predict and adjust nanoparticle properties in situ [64].

Integrating large and complex biological datasets generated from LoC platforms also remains a significant challenge as microfluidic technologies increasingly enable high-throughput and multiplexed analyses. Modern LoC systems can produce large volumes of heterogeneous data, including imaging outputs, biochemical assay results, genomic or proteomic readouts, and real-time sensor measurements. However, this capability also introduces challenges in data management, standardization, and interpretation. The lack of standardized data formats and analytical pipelines complicates the integration and comparison of datasets generated by different microfluidic devices and experimental protocols. Consequently, effective integration of LoC-generated datasets remains an interdisciplinary challenge spanning microfluidic engineering, bioinformatics, and data science, highlighting the need for standardized workflows and scalable computational tools to fully exploit high-throughput microfluidic technologies in biomedical research and nanomedicine [160,161].

Furthermore, as LoC systems advance toward clinical translation, the heterogeneity of patient-derived samples (e.g., blood, plasma, or mixed cell populations) and variability in biological inputs generate highly complex data streams [157]. These data must be processed, normalized, and interpreted with precision, necessitating dedicated software infrastructure, standardized data formats, and validated analytical protocols, many of which are still under development. As microfluidic technologies transition from research laboratories to real-world applications, the absence of harmonized data pipelines and regulatory data standards could hinder reproducibility, scalability, and eventual clinical acceptance [58,59].

### 5.5. Cost and Translation to Clinic

Economic and translational barriers remain substantial. Although microfluidic devices can reduce reagent consumption and offer precise control, manufacturing high-precision devices at scale, such as through injection molding or hot embossing, remains costly and demands design-for-manufacturability strategies [159].

Regulatory translation poses another major hurdle. LoC platforms intended for clinical or diagnostic use must comply with stringent regulatory frameworks (e.g., ISO standards and Good Manufacturing Practice). However, because many LoC devices are custom-designed for research applications, there is often no clear regulatory pathway, and standardized clinical validation workflows are not yet established for microfluidic-based nanoparticle systems. For LoC technologies to fulfil their promise in healthcare, alignment among researchers, manufacturers, regulators, and payors is essential; however, current incentives and economic models remain poorly coordinated [81].

Finally, integrating microfluidic-based NP production into existing pharmaceutical manufacturing or clinical pipelines is far from straightforward. High capital investment, the need for specialized expertise, and uncertainty regarding cost–benefit trade-offs (e.g., whether microfluidic manufacturing genuinely reduces cost per dose at scale) continue to slow adoption. However, bridging the gap between lab-scale systems and Good Manufacturing Practice (GMP) facilities still remains a significant challenge [151].

## 6. Emerging Trends and Future Perspectives

### 6.1. AI and Machine Learning in LoC–Nanomedicine Integration

Artificial intelligence (AI) and machine learning (ML) are increasingly being integrated into microfluidic systems to create “intelligent microfluidics” capable of optimizing NP synthesis, monitoring in-line performance, and adapting processes in real time [162]. Recent studies have demonstrated that coupling ML algorithms with microfluidic NP synthesis enables accurate prediction and fine-tuning of NP size, polydispersity, and surface charge using models such as decision trees, random forests, and neural networks [64] with, e.g., supervised ML models based on the Python module *scikit-learn* and artificial neural networks [163]. These data-driven approaches minimize the need for laborious trial-and-error exploration of parameter space and facilitate automated feedback loops for synthesis optimization [163].

AI is also advancing micro- and nanorobotic behavior within microfluidic environments, where algorithms such as deep learning, reinforcement learning, and swarm intelligence are used to control microrobot motion, collective dynamics, sensing, and task performance in biomedical microsystems [61]. Beyond NP synthesis, AI-accelerated high-throughput microfluidic platforms are increasingly employed for biomedical screening applications. For example, in drug discovery and diagnostic assays, ML models can analyze large datasets generated from parallel microfluidic channels, thereby reducing manual intervention and substantially improving experimental throughput [59].

Despite the rapid development of AI and ML and their applications in this area, significant challenges and bottlenecks remain in both technical and regulatory aspects. These are largely associated with (i) the reproducibility of microfluidic platforms, even when fabricated with high precision; (ii) data scarcity, as many ML models are currently trained on small or proprietary datasets; (iii) scalability limitations when translating microfluidic systems to industrial-scale manufacturing; (iv) limited explainability, as predictions generated by AI systems must be sufficiently interpretable to understand the mechanistic basis behind model recommendations; and (v) regulatory challenges in ensuring appropriate standards of transparency, reliability, and reproducibility [164,165].

### 6.2. Stimuli-Responsive Nanocarriers

The future of nano DDSs lies in the development of smart, stimuli-responsive nanocarriers capable of dynamically adapting to their physiological environment, including pH-sensitive polymers, redox-active systems, and ligand-triggered assemblies [166,167]. For example, pH-responsive polymeric nanoparticles based on materials such as poly(histidine), poly(β-amino esters), and chitosan derivatives can remain stable in the bloodstream (pH 7.4) but rapidly destabilize and release their payload in the acidic tumor microenvironment (pH 6.5–6.8) or within endosomes and lysosomes (pH 5.0–6.0) [166,168]. Similarly, redox-responsive nanocarriers incorporating disulfide bonds or thioketal linkages can selectively degrade in the cytosol, where glutathione concentrations are several hundred times higher than in extracellular fluids, thereby promoting intracellular drug release [169]. Ligand-triggered systems represent another emerging approach, where nanoparticles decorated with targeting ligands, such as folate, transferrin, antibodies, or peptide motifs, bind to overexpressed receptors on cancer cells and initiate receptor-mediated endocytosis, ensuring highly selective drug delivery [23].

When integrated with microfluidic platforms, responsive nanomaterials can be designed and tested under highly controlled and reproducible conditions [166], while enabling systematic evaluation of nanocarrier responses to physiological stimuli. For example, tumor-on-chip and organ-on-chip models can reproduce gradients in pH, oxygen, and nutrients typical of solid tumors, making them useful for testing pH-responsive liposomes or polymeric micelles. Similarly, microfluidic vascular models can simulate blood-flow-related shear stress to assess nanoparticle stability, adhesion, and release behavior under realistic conditions [170].

Integrating AI and ML with microfluidic experimentation is expected to accelerate the development of next-generation nano DDSs. AI models can analyze screening data and predict how formulation parameters, such as polymer composition, molecular weight, crosslinking density, ligand affinity, or surface charge, affect responsiveness to environmental triggers. These tools can help identify optimal polymer blends, improve ligand–receptor targeting efficiency, and potentially enable patient-specific nanoparticle designs tailored to disease characteristics such as tumor redox state or receptor expression [171].

### 6.3. 3D Printing and Hybrid Microfluidic Systems

Additive manufacturing is transforming the fabrication of LoC devices. For instance, direct laser writing (DLW) has recently been employed to nano-print PDMS microvessels with precisely defined architectures, such as controlled tortuosity and porosity, directly on top of 3D-printed chip substrates [172]. Furthermore, hybrid microfluidic systems that combine 3D-printed modules for fluid handling with microscale features fabricated via DLW are gaining significant traction. As highlighted by Young et al. [173], microfluidic components can be printed separately using DLW-enabled 3D printing and subsequently interfaced with larger-scale capillaries or chip modules, offering enhanced design flexibility and modularity.

Rapid prototyping of multiphase flow devices is also becoming increasingly accessible. A recent study demonstrated that 3D printing can be used to create microfluidic systems for emulsion generation, modular architecture, and on-demand design adaptation [174]. The convergence of 3D printing and microfluidics is poised to enable bespoke, patient-specific micro drug delivery system (micro DDS) manufacturing platforms, such as compact “microfactories” capable of batch-producing nanomedicines on demand.

### 6.4. Self-Powered and Portable LoC Systems

Wearable, self-powered LoC systems represent a rapidly emerging frontier in biomedical microengineering. Recent developments integrate microfluidic platforms with microneedle arrays to produce smart, wearable devices capable of both sampling biofluids, such as interstitial fluid, sweat, or tears, and delivering therapeutic nanocarriers or drugs in response to physiological cues [175].

These closed-loop systems combine biosensing, data analytics, and actuation within a single miniaturized platform. Embedded microfluidic circuits continuously sample and analyze biomarkers in real time, potentially supported by on-board AI for predictive analytics, and subsequently trigger nanoparticle or drug release via microvalves, electrochemical actuators, or thermoresponsive mechanisms. Such self-contained, low-power architectures enable autonomous operation without external power sources, often leveraging energy harvesting from motion, body heat, or biochemical gradients.

By enabling continuous health monitoring and adaptive, feedback-controlled therapy, wearable LoC devices could revolutionize personalized medicine. They hold particular promise for chronic disease management, point-of-care diagnostics, and remote therapeutic delivery, extending clinical capabilities beyond traditional healthcare settings.

### 6.5. Translation to Clinical and Pharmaceutical Practice

Translating LoC-based nano DDS platforms into clinical use will require overcoming substantial regulatory, manufacturing, and validation barriers. These challenges include establishing standardized fabrication protocols, ensuring batch-to-batch reproducibility, and demonstrating long-term biocompatibility and device reliability under clinical conditions.

Modular, 3D-printed microfluidic systems produced through scalable manufacturing techniques, such as injection molding or hybrid additive-subtractive methods, offer a potential pathway to reduce production costs and facilitate regulatory approval by promoting design standardization and traceability. In parallel, AI-enhanced microfluidic manufacturing introduces predictive modeling and closed-loop feedback control to ensure process reproducibility and quality assurance; however, regulatory bodies will need to develop clear frameworks for validating such data-driven and adaptive manufacturing systems.

Furthermore, the emergence of portable and wearable microfluidic–nanomedicine devices may redefine clinical workflows by enabling home-based dosing, continuous therapeutic monitoring, and point-of-care nanotherapy. Nevertheless, robust clinical trials, comprehensive safety evaluations, and interoperability standards will be essential to establish efficacy, patient safety, and public trust in these next-generation therapeutic technologies.

### 6.6. Toward Fully Integrated Nano-Pharmaceutics Platforms

The field is steadily progressing toward fully integrated nano-pharmaceutics platforms, comprehensive systems that merge AI-guided formulation, continuous microfluidic synthesis, real-time quality control sensing, and on-chip biological evaluation (e.g., OoC assays) within a single, automated pipeline.

Such integrated platforms could enable personalized nanomedicine, wherein patient-derived cells are incorporated into OoC models to test bespoke nano DDS formulations produced on demand by microfluidic modules. AI algorithms could then provide real-time feedback and adaptive optimization based on biological performance and formulation outcomes.

As data streams from formulation, processing, and biological evaluation become increasingly interconnected, these systems may generate comprehensive, multimodal datasets encompassing formulation parameters, pharmacodynamic responses, and safety profiles. The result would be a closed-loop, self-optimizing manufacturing engine capable of continuously refining nano DDS designs, paving the way toward autonomous, precision-driven nanotherapeutic development.

### 6.7. Regulatory Considerations for Microfluidic-Produced Nanomedicines

Regulatory considerations represent a critical factor in the clinical translation of nanomedicines produced using microfluidic technologies. Although microfluidic platforms enable highly controlled nanoparticle synthesis with improved reproducibility and batch-to-batch consistency compared with conventional bulk methods, regulatory agencies such as the FDA and EMA still evaluate these products under existing pharmaceutical regulatory frameworks rather than microfluidics-specific guidelines. As a result, developers are required to demonstrate rigorous characterization of key physicochemical attributes, including particle size distribution, surface properties, drug loading efficiency, and stability, to ensure safety, efficacy, and reproducibility during manufacturing and scale-up [176,177].

Despite the advantages of microfluidic manufacturing, several regulatory challenges remain. A major limitation is the absence of harmonized international standards for nanomedicine characterization, toxicity assessment, and quality control, which can complicate regulatory approval and slow clinical translation. Additionally, the complex interactions between nanoparticles and biological systems often require extensive preclinical evaluation to address concerns related to biodistribution, immunogenicity, and long-term safety. Consequently, regulatory agencies and researchers have emphasized the need for standardized analytical methods, quality-by-design approaches, and clearer regulatory guidelines to facilitate the safe development and commercialization of nanomedicine products generated through advanced manufacturing technologies such as microfluidics [178,179].

## 7. Conclusions

LoC platforms are rapidly transforming the landscape of nanomedicine by enabling precise, continuous, and highly controlled synthesis, characterization, and screening of nano drug delivery systems (DDSs). Across the review, we highlight how microfluidics provides unparalleled control over mixing, shear stress, reaction kinetics, and particle formation, leading to nanoparticles (NPs) with improved monodispersity, reproducibility, and tunability compared with conventional batch methods. Meanwhile, integrated sensing, organ-on-chip (OoC) modules, and real-time analytical tools allow for mechanistic insight into drug release, pharmacokinetics, and biological response, capabilities that traditional systems cannot match.

Despite impressive progress, key challenges remain, such as material biocompatibility, production scaling-up, standardization across fabrication methods, data integration, and regulatory translation, all of which remain barriers to industrial and clinical applications. Nonetheless, the convergence of LoC engineering with AI, smart materials, 3D printing, and automation is accelerating the development of next-generation nano-pharmaceutics platforms. Together, these technologies point toward a future where nanomedicines can be designed, synthesized, tested, and optimized entirely on-chip, enabling personalized therapeutics and significantly reducing development timelines.

The synergy between LoC technologies and nano DDSs marks a paradigm shift in drug delivery and personalized medicine. By merging precision engineering, systems biology, advanced materials, and computational intelligence, future nano-pharmaceutics platforms will deliver unprecedented control over therapeutic design and evaluation. Continued interdisciplinary collaboration, spanning microfluidics, nanotechnology, machine learning, and clinical science, will be essential to unlocking the full translational potential of these integrated systems.

## Figures and Tables

**Table 1:** Performance metric comparison between LoC/microfluidic platforms and conventional batch systems in the synthesis of nano drug delivery systems.

Performance Metric	Conventional Batch (e.g., Stirred Tank)	LoC/Microfluidic Systems	Significance in DDS Application
Particle Size (nm)	10–300 nm (high variance)	20–120 nm (highly tunable)	Smaller and more uniform nanoparticles enhance cellular uptake. [64,65]
Polydispersity Index (PDI)	0.2–0.5 (broad distribution)	<0.1–0.2 (monodisperse)	Predictable PK/PD profiles. [66,67]
Mixing Time (*τ_mix_*)	Seconds–minutes	Milliseconds	Rapid mixing prevents uncontrolled particle growth. [68]
Flow Rate (mL/min)	N/A (static/bulk)	0.01–10 mL/min	High precision over residence time. [69]
Shear Stress	Non-uniform	Controlled/predictable	Regulates impacts of shear stress on biologics. [70]
Encapsulation Efficiency (%)	40–70%	80–95%	Improved drug loading and reduced waste of expensive APIs. [71]
Batch-to-Batch Variability	High (operator-dependent)	Minimal (digital/automated)	Essential for clinical/regulatory approval. [64]

## Data Availability

Data sharing is not applicable to this article as no datasets were generated or analyzed during the current study.
